# Histological and Immunohistochemical Findings of an Extremely Rare Multi‐Organ Metastasis of Fibrosarcoma in a Holstein Cow

**DOI:** 10.1002/vms3.70567

**Published:** 2025-08-11

**Authors:** Abdolrasoul Namjou, Reza Kheyrandish, Akbar GholamZadeh, Fatemeh Zahra Kiani, Sadegh Shirian

**Affiliations:** ^1^ Department of Veterinary Shahrekord Branch Islamic Azad University Shahrekord Iran; ^2^ Department of Pathology School of Veterinary Medicine Kerman University Kerman Iran; ^3^ Department of Pathobiology School of Veterinary Medicine Shahrekord University Shahrekord Iran; ^4^ Shiraz Molecular Pathology Research Center Dr. Daneshbod Path Lab Shiraz Iran

**Keywords:** cow, fibrosarcoma, immunohistochemistry, spindle cell neoplasms

## Abstract

**Introduction:**

Fibrosarcomas are most frequently encountered in senior dogs and cats, but they have also been documented in various other species such as alpacas, llamas, ferrets, cows, sheep, goats and horses. Macroscopic, microscopic and immunohistochemical features of a very rare and unusual case of fibrosarcoma with metastasis to several visceral organs in a 4 year‐old Holstein cow is reported for the first time.

**Case summary:**

A cow with a history of decreased appetite, decreased milk production and chronic mastitis was presented to Abarkuh abattoir in Yazd province 4 months post‐parturition. The routine examination and systematic meat inspection revealed multi‐nodular focal masses, yellowish‐white in colour with firm consistency and clear borders on the surface and depth of the liver, kidney and heart, as well as in the parenchyma of both lungs. The masses showed a smooth surface without scars, bleeding and necrotic lesions. The tissue samples (1 × 1 × 1 cm^3^) were taken from the masses. Tissues were fixed in 10% buffered formalin and stained with haematoxylin and eosin, Masson's trichrome, and immunohistochemical staining using anti‐desmin, smooth muscle antibody (SMA), vimentin and CD34 antibodies. The histopathological evaluation of the masses showed spindle‐shaped cells, pleomorphic nuclei with mitotic figures and little cytoplasm. Masson's trichrome staining showed variable collagen fibres between abnormal fibroblasts. Immunohistochemical staining showed positive immunoreactivity for vimentin and negative for desmin, SMA and CD34 antibodies that are useful to distinguish this type of cancer from other spindle cell sarcomas. On the basis of the most recent literature search, this is the first report of multi‐organ metastasis of fibrosarcoma in a Holstein cow in the world.

## Graphical abstract

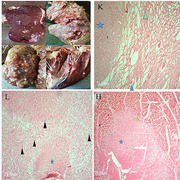

## 1 Introduction

Soft tissue sarcomas (STS) represent malignant tumours originating from connective tissues (Liegl‐Atzwange 2020). In fibrosarcoma, the predominant cell type is fibroblasts, which, when undergoing uncontrolled division and transformation, exhibit a significant potential for local growth, invasion and recurrence post‐surgery, although their metastatic capacity is relatively low (Wilson 2017; Augsburger et al. 2017). Fibrosarcomas are most frequently encountered in senior dogs and cats (Goldschmidt and Hendrick 2002), but they have also been documented in various other species such as alpacas, llamas, ferrets, cows, sheep, goats and horses (Pesato et al. 2018).

Fibrosarcoma can be induced by carcinogens, including subcutaneous vaccine injections against rabies and feline leukaemia virus, which have been linked to an increased incidence of sarcomas at vaccination sites (Woodward 2011). Other causative factors encompass microchip implants (Vascellari et al. 2006), chronic inflammation (Conlon et al. 2021), ionizing radiation, cytostatic activity and genetic disorders associated with p53 or RB113 gene mutations in mammals (Wilson 2017), as well as retroviral infections (Hartmann 2012). Fibrosarcomas manifest in three forms: congenital, juvenile and adult (Damodaran et al. 1974; Hultgren et al. 1987; Rosiers et al. 2020; Orr 1984; Hamali and Ashrafihelan 2010; Musal et al. 2007; Britt et al. 1998). Cattle, as hosts for these uncommon mesenchymal tumours, can develop fibrosarcomas in various body regions, particularly the reproductive organs (Orr 1984; Yeruham et al. 1999). This susceptibility leads to infertility, dystocia and economic losses (Orr 1984; Hamali and Ashrafihelan 2010; Musal et al. 2007; Britt et al. 1998; Yeruham et al. 1999). In this report, the macroscopic, microscopic and immunohistochemical features of an extremely rare metastatic fibrosarcoma with an unknown primary site, accompanied by metastases to multiple visceral organs in a Holstein cow is presented. Although this tumour type has been previously reported in Holstein cows in Iran (Shokrpoor et al. 2023) and other countries (Braun et al. 2001), this case stands as the first documented instance of metastatic fibrosarcoma in a Holstein cow in the world.

## 2 Case Presentation

**FIGURE 1:**
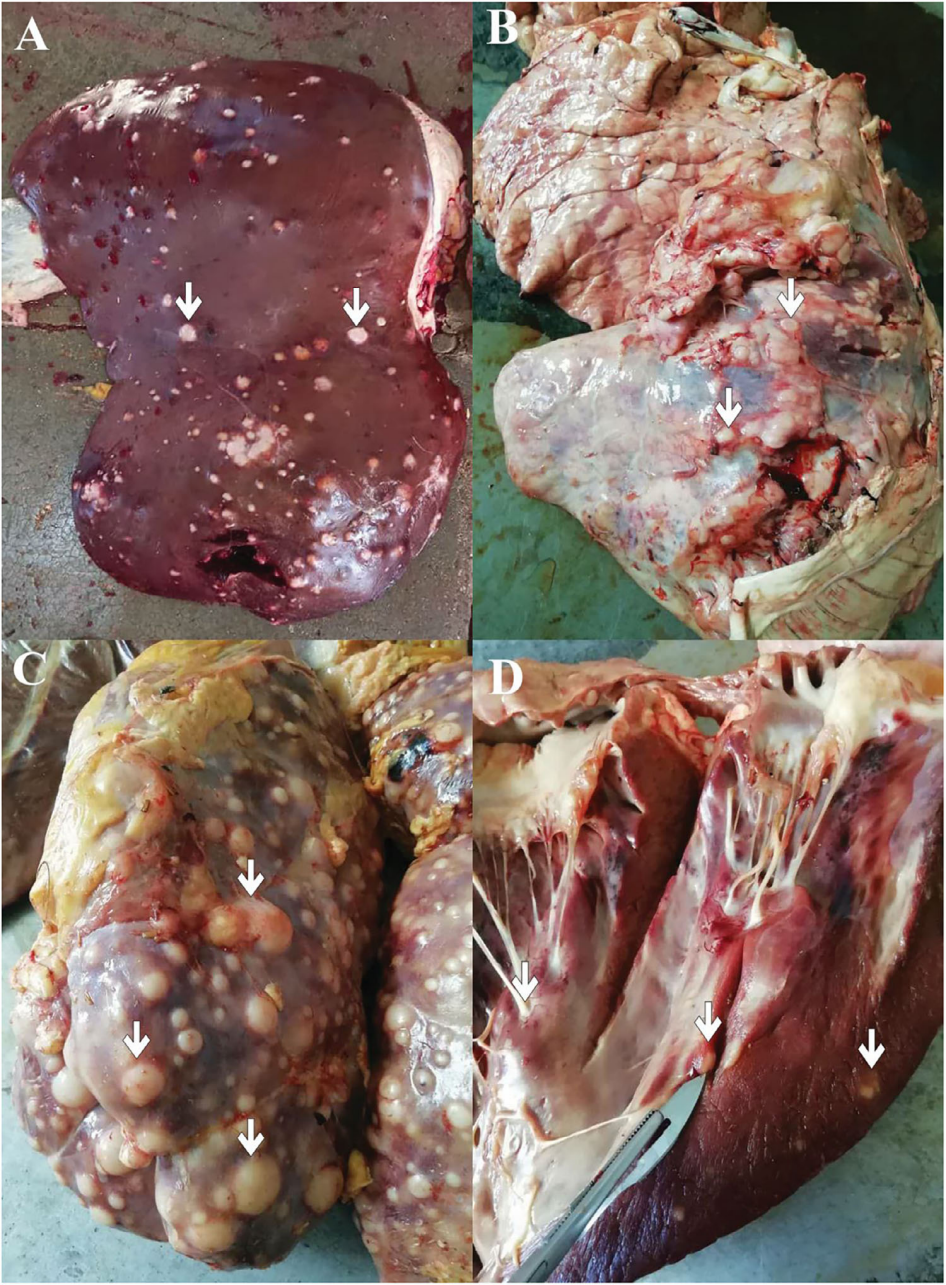
White nodular lesions of metastatic fibrosarcoma (white arrows) on the surface of the liver (A), lung (B), kidney (C) and heart (D).

A 4‐year‐old Holstein cow with a history of poor appetite, decreased milk production, chronic mastitis and 4 months postpartum was sent to an abattoir. Gross examination of the carcass at the abattoir revealed raised and nodular, coalescing masses with diameters of 1–3 cm randomly scatter in the liver, kidney, heart and muscles. Widespread lesions with firm consistency and yellowish‐white to grey cut surface in the lung parenchyma were observed (Figure 1). Samples from the affected organs were fixed in 10% buffered formalin. The samples were then processed for haematoxylin and eosin, Masson's trichrome, and immunohistochemical staining. Paraffin Sections with 3‐µm thickness were used for the immunohistochemical staining. After deparaffinized using xylol, the sections were rehydrated and treated with 3% hydrogen peroxide solution for 10 min at room temperature. The antigen retrieval was conducted with microwaving using a 10‐mmol/L concentration of citrate buffer (pH 6.0). The primary antibodies, including anti‐vimentin, desmin, smooth muscle antibody (SMA) and CD34 were applied for 1 h (diluted 1:200). The detection system used was Envision‏ (DakoCytomation, Glostrup, Denmark) and developed with diaminobenzidine (DakoCytomation) as previously reported (Vasconcelos et al. 2023). 3,30‐Diaminobenzidine hydrogen peroxide was applied as the chromogen, and haematoxylin was used as the counterstain. All stained sections were evaluated by three pathologists under a light microscope.

**FIGURE 2:**
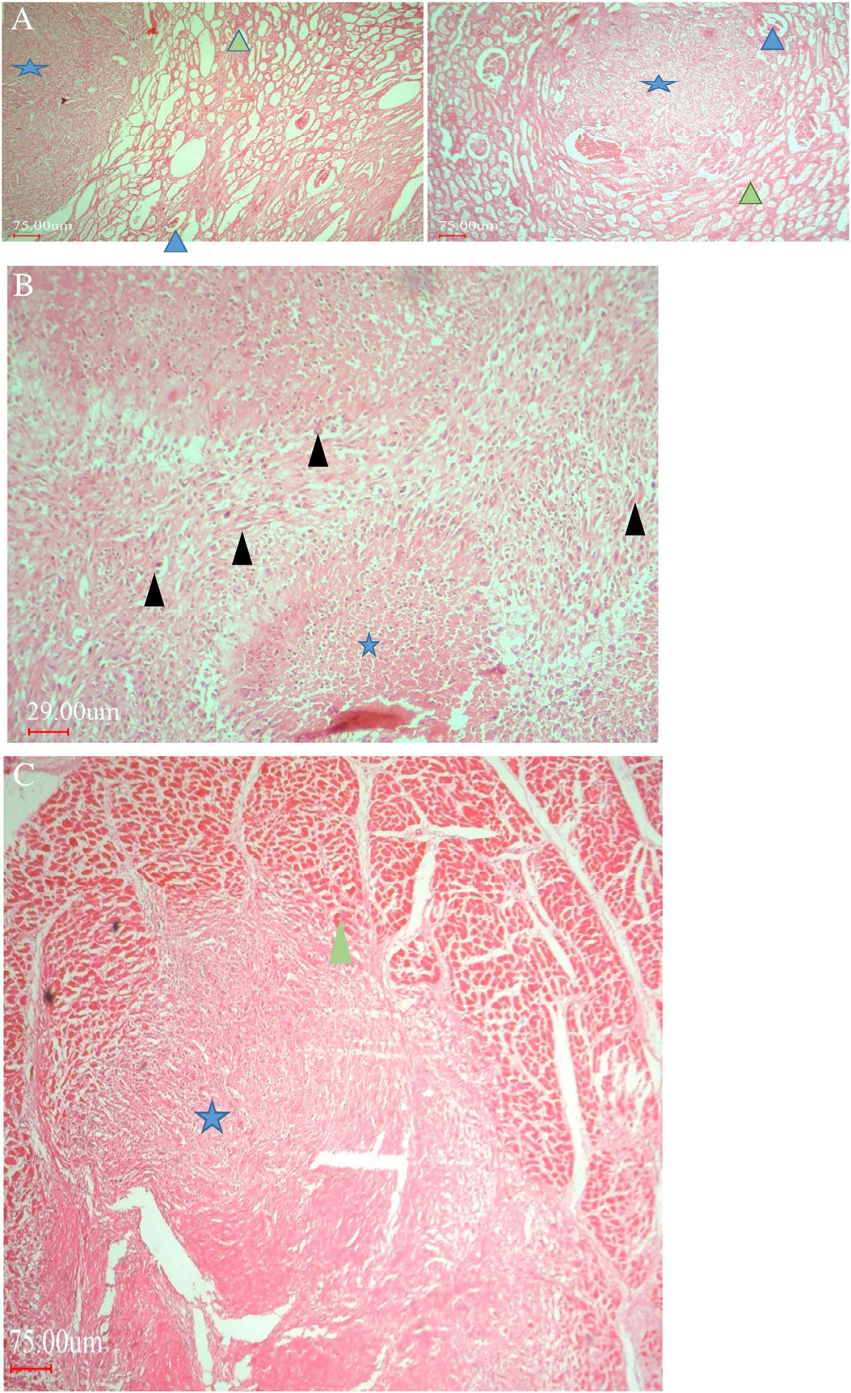
(A) Microscopic figures of a metastatic fibrosarcoma in kidney showing bundles and streams of neoplastic spindle cells and collagen fibres with typical whorled pattern (star), leading to pressure atrophy (blue arrow head), acute tubular necrosis (green arrow head) and destructed glumerols, and bizarre cells, ×200, scale bar 75 µm. (B) Microscopic figures of a metastatic fibrosarcoma in liver showing pleomorphic with hyperchromatic nuclei neoplastic spindle cells with multi‐nucleoli (black arrow heads) associated with sever necrosis (star), ×400, scale bar 29 µm. (C) Microscopic figures of a metastatic fibrosarcoma in heart showing pleomorphic with hyperchromatic nuclei neoplastic spindle cells and collagen fibres with typical whorled pattern (star) and necrotic maucles (green arrow head), ×200, scale bar: 75 µm.

**FIGURE 3:**
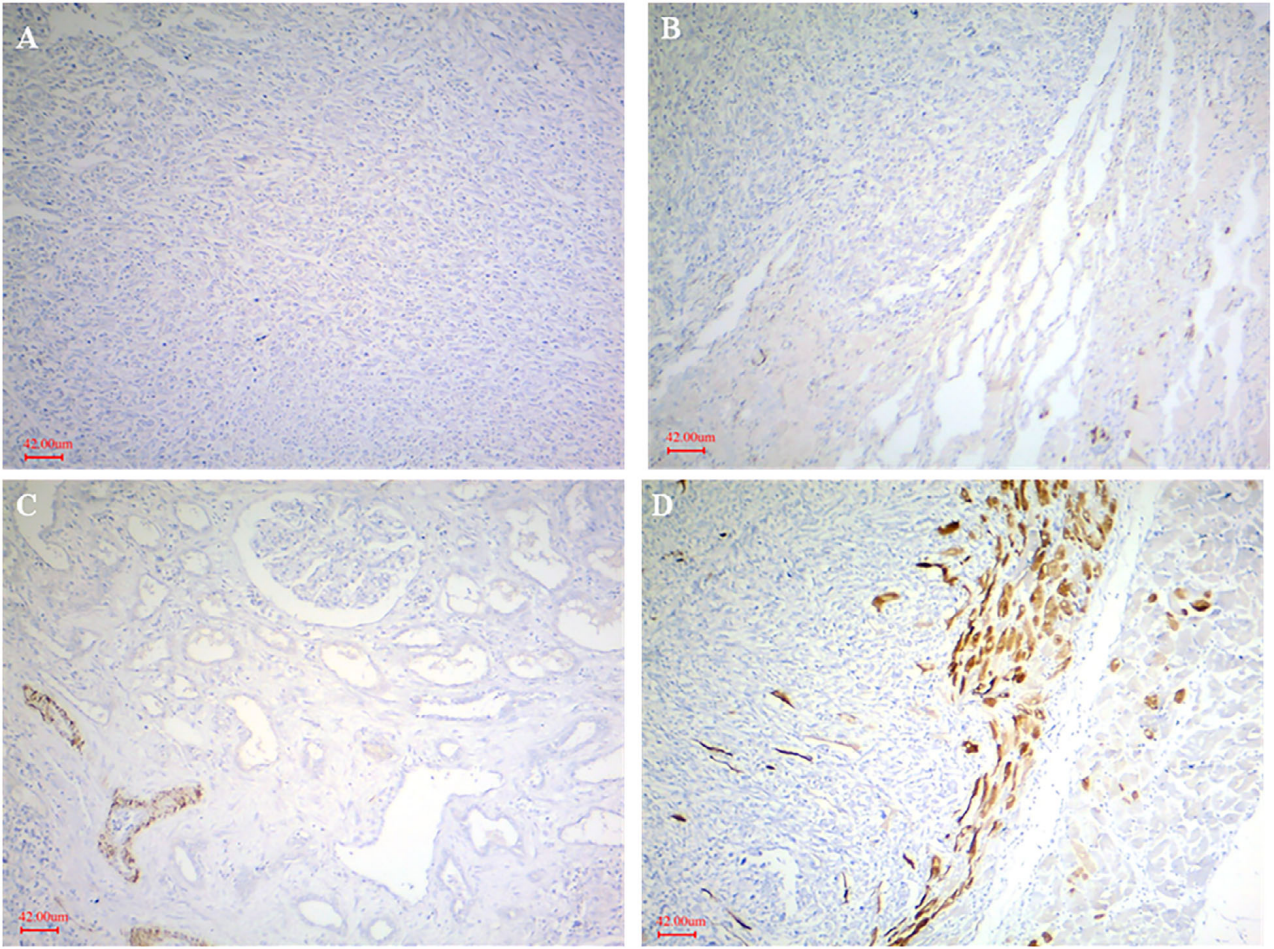
Negative immunoreactivity for desmin staining of the tumour in liver (A), lung (B), kidney (C) and heart (D) tissues. Only the muscle fibres of heart (brown colour) show positive staining for desmin, immunohistochemical staining, ×100, scale bar: 42 µm.

**FIGURE 4:**
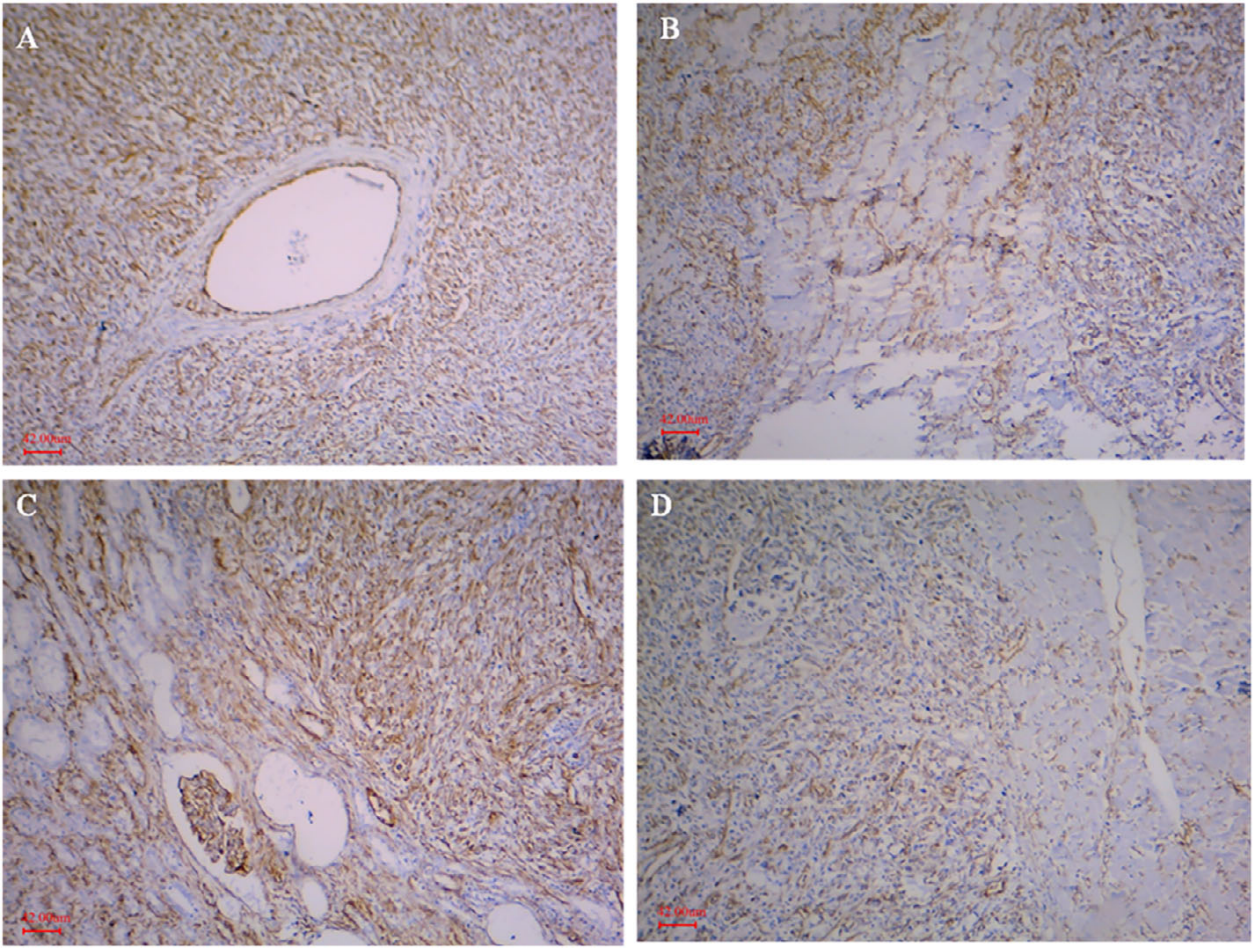
Positive immunoreactivity for vimentin staining in liver (A), lung (B), kidney (C) and heart (D) for vimentin, immunohistochemical staining, ×100, scale bar: 42 µm.

Histopathologically, the liver, kidney and heart lesions showed the proliferation of pleomorphic spindle‐shaped cells. The cells were characterized by hyperchromatic nuclei and little cytoplasm in interwoven and a herring pattern (Figure 2). The high mitotic index was seen with objective lens magnification of 400. Masson's trichrome staining was performed to definitively identify collagen fibres in polygonal nucleus tumour cells. Immunohistochemical staining showed a positive reaction for vimentin expression, but the reaction of the cytoplasm of neoplastic cells was negative for desmin, SMA and CD34 (Figures 3 and 4).

## 3 Discussion

In this study, histological and immunohistochemical features of an extremely rare metastatic fibrosarcoma affecting several organs in a Holstein cow is reported for the first time. Squamous cell carcinomas is the most frequent carcinoma in ruminants (Soleimanpour et al. 2011). Fibrosarcoma is a tumour of mesenchymal origin that is composed of malignant fibroblasts, which has been rarely reported to metastasize to internal parenchymatous organs (Hartmann 2012). The macroscopic characteristics of metastatic fibrosarcoma found in this case were similar to those previously reported (Orr 1984). Macroscopic findings and histopathologic evaluation with routine haematoxylin and eosin staining method are not enough to differentiate between fibrosarcoma and other spindle cell cancers. Immunohistochemical staining or protein tumour markers are required to distinguish fibrosarcoma from other malignant spindle cell tumours (Vasconcelos et al. 2023; Bahrami and Folpe 2010; Cozzolino et al. 2013; Miettinen 2014; Meuten 2007). Microscopic studies with special staining and immunohistochemistry of the reported tumour led to the diagnosis of fibrosarcoma (Findley et al. 2014). Although the primary origin of the tumour was not clearly determined, based on the history of chronic mastitis and decreased milk production, the possibility of a fibrosarcoma tumour in the udder cannot be excluded (Piva et al. 2017).

Fibrosarcoma can originate from the soft tissue or bone. It can develop into the endosteum or periosteum, then involve the adjacent soft tissue (Pelmus et al. 2002) and other organs. The histological grading of fibrosarcoma in humans depends on factors such as mitotic activity, cell density, cell differentiation, nuclear pleomorphism, necrosis rate and collagen matrix production rate (Gown 2013; Kaur et al. 2022). On the basis of the malignancy, fibrosarcomas are classified into low and high grade cancers (Augsburger et al. 2017). Low‐grade fibrosarcoma shows spindle cells, which are arranged in small to moderate cellular fascicles with a herringbone appearance (Gown 2013). Low nuclear pleomorphism and rare mitosis with collagenous stroma (Mark et al. 1991) show a low‐grade fibrosarcoma, which rarely invades deep tissues and causes distant metastases (Hartmann 2012). In contrast, the high‐grade lesion shows a high nuclear pleomorphism, increased cellularity and abnormal mitosis, with a nuclei which can be spindle‐shaped, oval or round (Augsburger et al. 2017). High‐grade is usually more invasive and carries a worse prognosis (Orr 1984).

Differential diagnosis of fibrosarcoma with other spindle cell tumours is performed by specific staining, detection of collagen fibres and immunohistochemistry. In this case report, after review of the histological sections from all tissue samples, the collagen fibres between abnormal fibroblast was confirmed, which is consistent with previous studies (Kaur et al. 2022). Fibrosarcoma should be differentiated from peripheral nerve sheath tumours and leiomyosarcomas (Hartmann 2012).

In addition, using the antibody reaction, immunohistochemistry plays an important role in the diagnosis (Chang and Kessler 2008). Immunohistochemistry was used to rule out other spindle cell tumours such as leiomyosarcoma, myofibrosarcoma, rhabdomyosarcoma and hemangiosarcoma. Desmin and SMA are known as the most common markers for myogenic differentiation. CD34 is a helpful marker in the diagnosis of spindle cell angiosarcoma (Miettinen 2014). Positive staining with vimentin indicates that the origin of spindle‐shaped mesenchymal cells is fibrosarcoma. Other spindle‐shaped cell tumours such as leiomyosarcoma react positively with both vimentin and desmin (Pesato et al. 2018).

Vimentin is a protein tumour marker that is characteristic of mesenchymal cell origin and is frequently the only positively stained marker in the diagnosis of fibrosarcomas (Couto et al. 2002). Histologically, fibrosarcoma shows higher cellularity with less collagen fibre density than fibroma. Moreover, atypical cells, abnormal mitotic divisions, marked increase in cells and other features of anaplasia may account with high malignancy and metastasis to internal organs (Hartmann 2012).

The results of immunohistochemistry showed that neoplastic fibroblast cells can be distinguished from the tumours of myofibroblasts and smooth muscle cells. The negative reaction of SMA and desmin should be considered a reason for the absence of myofibroblast lineage. In the immunohistochemical evaluation of the present tumour, the neoplastic cells were negative for CD34 protein, which is a useful marker for the diagnosis of vascular endothelial cell tumours. Currently, immunohistochemistry markers are not only used for the diagnosis and prognosis of tumours but also for management and specific treatment decisions (Parham 2015). To the best of the author's knowledge, this is the first report of metastatic fibrosarcoma to internal organs in cattle worldwide. Fibrosarcoma appears to progress rapidly, and due to vital organ involvement, carries a guarded prognosis. Unfortunately, the primary origin of the tumour was not clearly determined.

## 4 Conclusion

Although fibrosarcomas have previously been reported in animals worldwide, this is the first report describing a Holstein cow with metastatic neoplasia to multiple organs. Macroscopic and microscopic features, as well as positive and negative immunoreactivity for spindle cell tumour markers, were described.

## Author Contributions

**Abdolrasoul Namju**: writing – original draft, methodology. **Reza Kheyrandish**: methodology. **Akbar GholamZadeh**: methodology. **Fatemeh Zahra Kiani**: methodology. **Sadegh Shirian**: conceptualization, methodology, writing – original draft. All authors contributed to diagnosis and read and approved the MS.

## Ethics Statement

The authors have nothing to report.

## Conflicts of Interest

The authors declare no conflicts of interest.

## Data Availability

The data are available via contacting with corresponding authors.
